# [Retracted] Prognostic and predictive roles of microRNA-411 and its target STK17A in evaluating radiotherapy efficacy and their effects on cell migration and invasion via the p53 signaling pathway in cervical cancer

**DOI:** 10.3892/mmr.2026.13886

**Published:** 2026-04-22

**Authors:** Wei Wei, Cun Liu

Mol Med Rep 21: 267–281, 2020; DOI: 10.3892/mmr.2019.10826

Following the publication of the above paper, it was drawn to the Editor's attention by a concerned reader that, regarding the flow cytometric plots shown in Fig. 11A on p. 279, these plots appeared to show similar groupings of dots, which would not have been anticipated if these experiments had been performed discretely under different experimental conditions, suggesting a fundamental flaw either in the way in which these experiments were performed or in how the results were outputted.

The Editor of *Molecular Medicine Reports* has decided that this paper should be retracted from the Journal on account of a lack of confidence in the presented data. The authors were asked for an explanation to account for these concerns, but the Editorial Office did not receive a reply. The Editor apologizes to the readership for any inconvenience caused.

